# Retinal myeloid cells regulate tip cell selection and vascular branching morphogenesis via Notch ligand Delta-like 1

**DOI:** 10.1038/s41598-019-46308-3

**Published:** 2019-07-05

**Authors:** Fabian Haupt, Kashyap Krishnasamy, L. Christian Napp, Michael Augustynik, Anne Limbourg, Jaba Gamrekelashvili, Johann Bauersachs, Hermann Haller, Florian P. Limbourg

**Affiliations:** 1 Vascular Medicine Research, Hannover Medical School, Hannover, Germany; 2 Department of Nephrology and Hypertension, Hannover Medical School, Hannover, Germany; 3 Department of Cardiology and Angiology, Hannover Medical School, Hannover, Germany; 4 Present Address: Department of Nuclear Medicine, Inselspital, Bern, University Hospital, University of Bern, Bern, Switzerland; 5 Present Address: Department of Plastic, Aesthetic, Hand and Reconstructive Surgery, Hannover Medical School, Hannover, Germany; 6 Present Address: Augenärzte am Meer, Bremerhaven, Germany

**Keywords:** Angiogenesis, Monocytes and macrophages, Angiogenesis

## Abstract

During angiogenesis, single endothelial cells (EC) specialize into tip cells that guide vessel sprouting towards growth factor gradients and instruct the adjacent vessel stalk. The balance between tip and stalk cells is regulated by endothelial Notch signalling through the expression of Notch ligand Delta-like 4 (Dll4) in tip cells, which suppresses a tip cell fate in adjacent stalk cells. Here we show, using genetic reporter and conditional deletion strategies, that myeloid cells regulate tip cell numbers and Dll4 expression via the Notch ligand Dll1 during vascular development in the retina. Dll1 is selectively expressed by a subpopulation of retinal myeloid cells, which progressively localizes to the sprouting vascular network. Conditional, myeloid-specific deletion of *Dll1* impairs endothelial Dll4 tip-stalk gradient resulting in an increase of endothelial tip cells and EC filopodia, accompanied by an increase in vascular density and branching. *In vitro*, co-culture of human EC with monocyte-derived macrophages induced Dll1 upregulation in macrophages and Dll4 upregulation and an endothelial tip cell signature in EC. Furthermore, culturing human EC on recombinant DLL1 induced endothelial Dll4 expression and a tip cell program, indicating that changes are Dll1-dependent. Thus, myeloid cells regulate tip cell fate and angiogenesis through expression of Notch ligand Dll1.

## Introduction

Angiogenesis is a coordinated process involving sprouting and remodelling of the vascular system. During angiogenesis, single endothelial cells (EC) specialize into tip cells (TC) with protruding filopodia that guide vessel sprouting towards growth factor gradients, such as the vascular endothelial growth factor (VEGF)^1^ and instruct the adjacent vessel stalk. TC migrate and fuse via their filopodia, while their adjacent EC – the stalk cells (SC) – do not migrate but rather proliferate and form the vessel lumen. The balance between TC and SC is a key regulator of angiogenesis^2,3^.

The development of the complex vascular network in the retina proceeds through several phases. The blood supply to the retina is initially supported by the hyaloid plexus. Upon the later developmental stages, blood vessels grow out from the central retinal artery at the optical nerve papilla and expand towards the inner retinal layer, while the hyaloid plexus regresses. In mice, the vascularization of the inner retinal layer takes place after birth. Around postnatal day 7 (p7) the vascular plexus reaches the outer margin of the retinal inner surface. At this time point vessel sprout out into deeper retinal layer and form a deep vascular plexus^4^.

Notch signalling is a key regulator of the TC-SC-balance. Notch is a family of highly conserved surface receptors, which interacts with surface bound ligands of the Delta-like (Dll) and Jagged/Serrate family via direct cell-cell-contact^5^. EC dynamically compete for the leading position at the angiogenic front by acquiring a TC phenotype. This is mediated by upregulation of the ligand Dll4 in TC, which activates Notch signalling and suppresses Dll4 expression in adjacent EC, inducing a SC phenotype^6^. *Dll4* loss-of-function leads to increased numbers of migrating tip cells^7,8^, resulting in EC hypersprouting and defective angiogenesis^9^. The Notch ligand Dll1 shows an expression pattern distinct from Dll4 and also regulates angiogenesis^10,11^. In the developing blood vessels of the retina, general heterozygous *Dll1* loss-of-function leads to impaired angiogenesis and defects in vascular branching morphogenesis^12^. While Dll4 is expressed by TC, Dll1 expression was absent in vascular endothelium, but instead observed in cells adjacent to the vascular layer in the retina^10,12^. The cell type expressing Dll1 remains unknown.

Retinal myeloid cells (RMC) are a heterogeneous population of resident microglia and monocyte-derived macrophages regulating angiogenesis. Microglia represent a resident macrophage population derived from circulatory cells of the primitive haematopoiesis in the embryonic yolk sac^13–15^, their origin is independent from late haematopoiesis^16^. These microglial progenitors enter the central nervous system (CNS) during embryonic development and proliferate within the CNS; even in the first postnatal days the number of microglia steadily increases^17,18^. RMCs from this early developmental stage are forming a population of tissue-resident myeloid cells, which is capable of self-renewal^16,19,20^. On the other hand, monocyte-derived macrophages are descendants of definitive haematopoiesis, originating either from the late-embryonic liver or from the bone marrow and are recruited into different tissues. Yet, retinal macrophages and microglia share many functional and phenotypic features, and macrophages can replace yolk sac derived resident microglia in injury models^21,22^.

Depending on context, myeloid cells can promote vessel maturation by chaperoning TC fusion or restrict angiogenesis by inhibiting vascular branching^23^.

Conditional deletion of *Notch1* in myeloid cells leads to an altered association with ECs at the angiogenic front and defective EC sprouting^24^. We here describe the effects of conditional myeloid deletion of *Dll1* on retinal angiogenesis.

## Results

### Myeloid cells express Dll1 during vascular development in the retina

**Figure 1:**
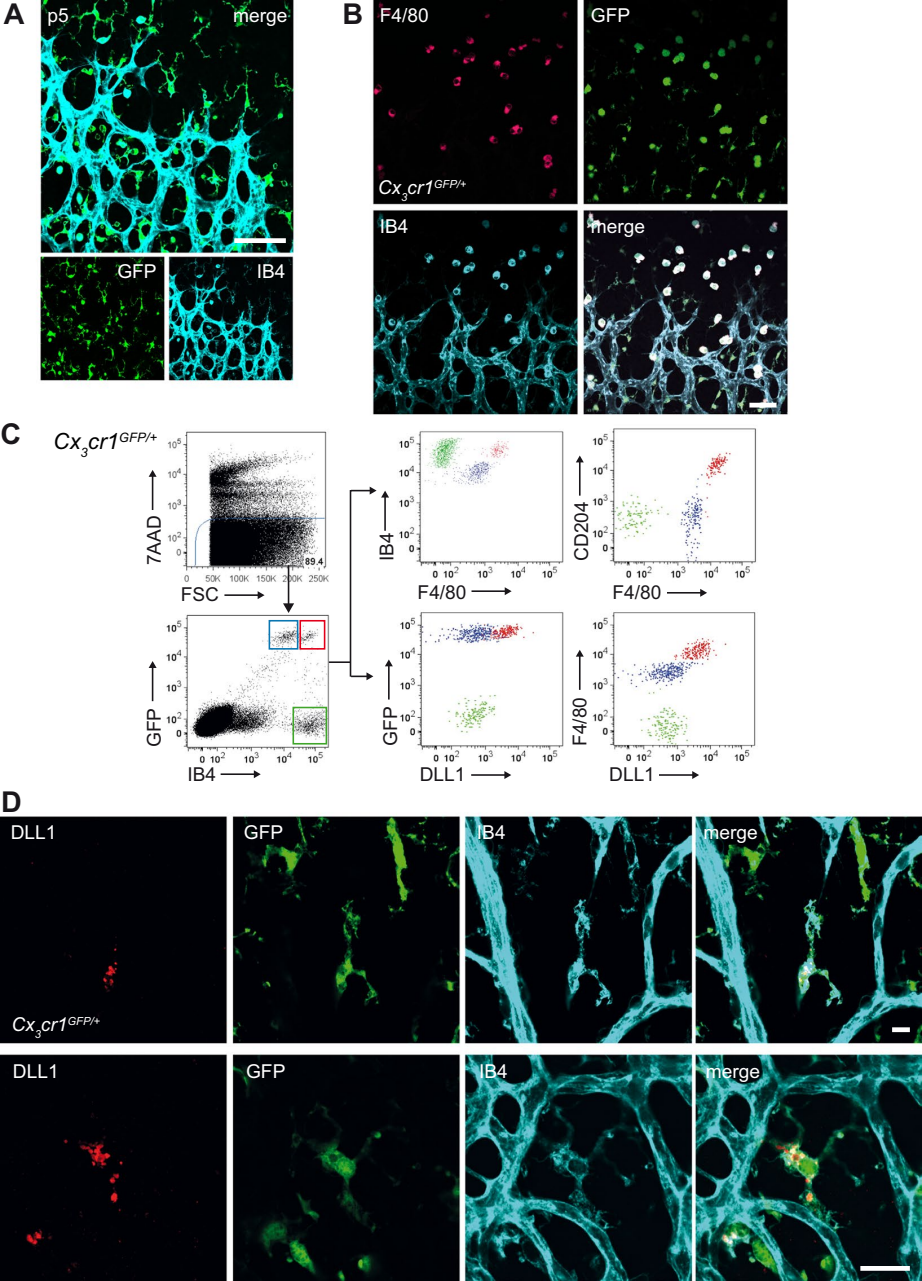
Identification and characterization of Dll1 expressing retinal myeloid cells. (**A**) Retina whole mount immunofluorescences of postnatal day 5 (p5) *Cx*3*cr1*^GFP/+^ mice (50X magnification; scale bar: 75 µm). Green fluorescent protein positive (GFP^+^) myeloid cells can be differentiated from GFP^−^ endothelial cells (EC), stained by IsolectinB4 (IB4). **(B)** Retina whole mount immunofluorescences of p5 Cx3cr1^GFP/+^ mice. The GFP^+^IB4^hi^ myeloid cells also stain high for F4/80. In addition, another population, which can be characterized as GFP^+^IB4^lo^F4/80^lo^ can be seen in the retina (50X magnification; scale bar 50 µm). The cells are in close association to the neighbouring tip cells (TC). (**C)** Whole retinas of p5 *Cx3cr1*^GFP/+^ mice were analysed by flow cytometry (FACS). Based on GFP and IB4, gating was performed on two GFP^+^ myeloid subpopulations (red and blue gate) – differentiated by their IB4 levels, and in addition on GFP^−^IB4^+^ endothelial cells (EC; green gate). Based on these gates, the myeloid populations were further characterized by their surface markers in comparison to each other and to the EC and analysed for DLL1 expression. (**D**) Retina whole mount immunofluorescences of p5 Cx3cr1^GFP/+^ mice. In contrast to the CX_3_CR_1_^+^IB4^lo^ population, the CX_3_CR_1_^+^IB4^hi^ myeloid cells within the superficial plexus showed intense DLL1 expression (top - 150X magnification; scale bar: 10 µm; bottom - 200X magnification; scale bar 20 µm).

To test the hypothesis that cells from the myeloid lineage express Dll1 near the developing vasculature, we studied *Cx*_3_*cr1*^GFP/+^ reporter mice, in which monocytes, macrophages and microglia, but not granulocytes and endothelial cells, express green fluorescent protein (GFP)^14,25^. We used GFP fluorescence to visualize myeloid cells in whole mount retina preparations, in addition to labelling with isolectin B4 (IB4), which stains EC and myeloid subpopulations (Fig. 1A)^13,23^. By confocal microscopy, GFP^+^ cells constituted a heterogeneous population, found in the superficial layer of the retina containing the developing vascular plexus, that display a unique spatial relationship to angiogenic tip cells, as described before^13,23,26^. IB4^lo^GFP^+^ cells had a small cell body but prominent ramified morphology, consistent with resident microglia, while IB4^hi^GFP^+^ cells had a large, spherical shape. Both populations were in direct contact with or adjacent to IB4^hi^GFP^−^ endothelial cells at the angiogenic front (Fig. 1A, Supplementary Fig. 1). While IB4^hi^GFP^+^ cells were only found in the superficial, vascular layer of the retina, IB4^lo^GFP^+^ cells were also present in the deep, avascular layers of the retina (Supplementary Fig. 1). Additional staining with the lineage marker F4/80^21,27^ showed that IB4^hi^GFP^+^F4/80^hi^ cells were in close proximity to EC in the angiogenic front (Fig. 1B).

We next employed flow cytometry to further define cell populations and characterize Dll1 expression on postnatal day 5 (p5). Compared to GFP^+^IB4^lo^ myeloid cells and GFP^−^IB4^hi^ EC, GFP^+^IB4^hi^ myeloid cells expressed high levels of F4/80 and scavenger receptor CD204^23,28^, a signature suggestive of monocyte-derived macrophages^21^. More importantly, flow cytometry of cell populations and confocal imaging revealed that only GFP^+^IB4^hi^F4/80^hi^ myeloid cells expressed DLL1 (Fig. 1C,D, Supplementary Fig. 2). Together, these results demonstrate that a subset of retinal myeloid cells expresses Dll1 during vascular development in the retina.

### Developmental kinetics of retina myeloid cell populations

**Figure 2:**
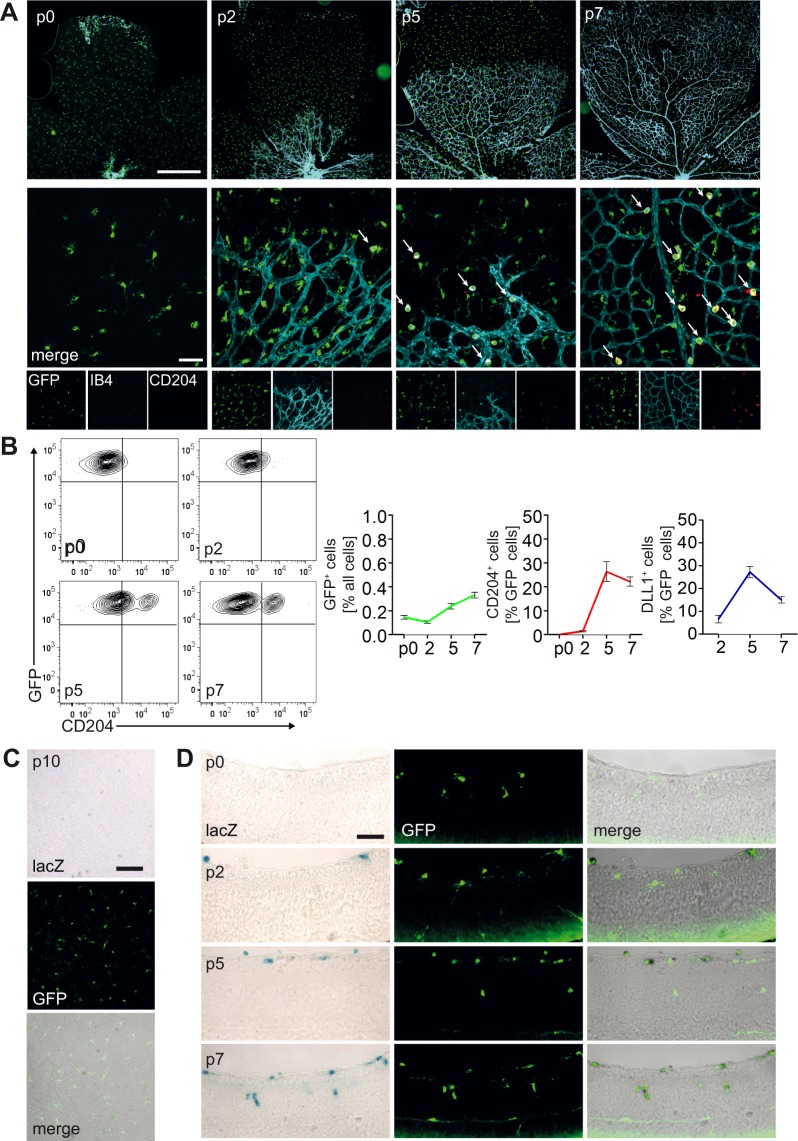
Population dynamics of myeloid cells in the developing retina. **(A)** Retina whole mount immunofluorescence images of p0 to p7 *Cx3cr1*^GFP/+^ mice as overviews (top; 5X magnification; scale bar: 500 µm) and confocal microscopy closeups (bottom; 50X magnification; scale bar: 50 µm). Retina were analysed for GFP (green) expression and stained with IB4 (blue), CD204 (Red) and GFP (green). **(B)** FACS analysis (left) of p0 to p7 Cx3cr1^GFP/+^ mice retinae (according to the gating strategy from Fig. 1B). Graph (right) depicting kinetics of myeloid GFP^+^ cell distribution, indicated as % of all cells: p0 0.15 ± 0.03, p2 0.10 ± 0.04, p5 0.24 ± 0.10, p7 0.33 ± 0.10 [mean ± SEM], CD204^+^ cells, indicated as % of GFP^+^ cells: p0 0.00 ± 0.00, p2 1.44 ± 0.68, p5 26.32 ± 10.26, p7 22.17 ± 3.34 [mean ± SEM] and DLL1^+^ cells, indicated as % of GFP^+^ cells: p2 6.56 ± 2.87, p5 27.23 ± 4.40, p7 34.97 ± 2.34 [mean ± SEM]. **(C)** β-galactosidase (β-Gal) staining of p10 LysMCre;Rosa^lacZ/lacZ^ x CX_3_CR_1_^GFP/+^ double reporter mice hindbrain cryosections (20X magnification; scale bar: 100 µm). **(D)** Fluorescence microscopy images of time course analysis of LysMCre;Rosa^lacZ/lacZ^ x *Cx*_*3*_*cr1*^GFP/+^ double reporter mice retina cryosections after β-galactosidase (β-Gal) staining (20X magnification; scale bar: 50 µm).

We next sought to characterize myeloid population dynamics in the retina by confocal imaging and flow cytometry. In contrast to the resident population of CD204^−^ myeloid cells, which was present before and during the initiation of vascular plexus formation (p0 to p2), the larger, CD204^+^ population was mostly absent from the retina at early stages of vascular development. However, concomitant to the phase of extensive vascular sprouting and remodelling, beginning after p2, there was an increase in the total GFP^+^ cell population in the retina (Fig. 2A,B). Furthermore, numbers of GFP^+^IB4^hi^CD204^+^ myeloid cells, and of Dll1^+^ myeloid cells steadily and significantly increased, localizing adjacent to and in between vascular sprouts.

We next characterized lineage relationships of retinal myeloid cells and investigated, whether myeloid subpopulations in the superficial vascular plexus can be discretely targeted by conditional recombination strategies. We employed compound genetic lineage tracing by combining the *Cx*_3_*cr1*^*GFP/+*^ reporter allele with a myeloid specific Cre-recombinase, *LysM*^*Cre*^ ^29^,which is restricted to cells of adult myelopoiesis including macrophages^30–32^. A β-galactosidase (β-gal) Cre-reporter allele (*R26R*^*lacZ*^) monitored Cre-mediated recombination in these compound reporter mice.

We first analysed p10 hindbrain sections for β-gal activity and GFP fluorescence, since this region is populated by yolk sac derived microglia^14^. Despite the presence of numerous GFP^+^ cells, there was no detectable β-gal activity (Fig. 2C), confirming, that yolk sac derived microglia are not targeted by *LysM*^*Cre*^. The retina at p0 was also populated by GFP^+^ cells, which were β-gal negative. However, starting with p2, there was progressive infiltration of the superficial, vascular layer of the retina by large, spherical cells that were β-gal^+^GFP^+^, while the β-gal^−^GFP^+^ subpopulation relocated into the deeper retinal layer (Fig. 2D). This suggest that retinal myeloid cells consist of distinct subpopulations and suggest mixing of resident microglia with recruited myeloid cell subsets^21^. Furthermore, it demonstrates distinct targeting of a subpopulation of retinal myeloid cells by LysM^Cre^, consistent with previous reports^13,30^.

### Myeloid cell Dll1 regulates vascular sprouting

**Figure 3:**
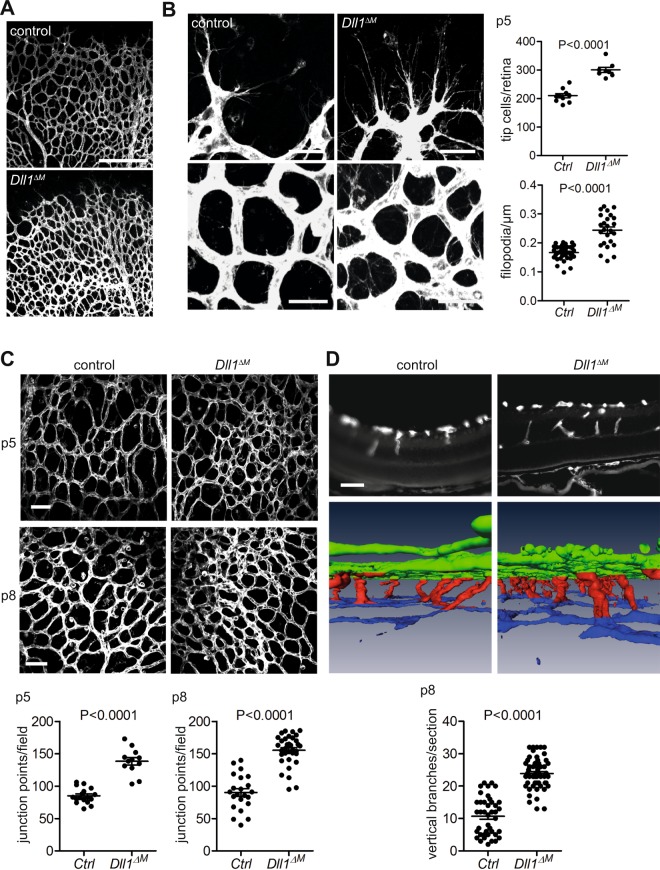
Loss of myeloid *Dll1* leads to excessive sprouting. (**A**) IB4 whole mount retina staining (10X magnification; scale bar: 250 µm) of p5 *LysM*^Cre/+^*Dll1*^flox/flox^ mice (Dll1^ΔM^) and *LysM*^+/+^*Dll1*^flox/flox^ littermates (control). (**B**) Closeup images (left top; 50X magnification; scale bar: 32 µm; left bottom; 100X magnification; scale bar 25 µm). Graph (right) depicting analysis of tip cells/retina (n = 10/8; p < 0.0001 unpaired students *t* test) and filopodes/µm (n = 13/5; p < 0.0001 unpaired students *t* test). **(C)** Images depicting analysis of connection points between control and Dll1^ΔM^ at p5 and p8 (50X magnification; scale bars: 50 µm; n = 6/7 for p5, n = 14/16 for p8; p < 0.0001 unpaired students *t* test). **(D)** Top: IB4 immunostained retina cryosection (p8; 40X magnification; scale bar: 50 µm); 3D reconstruction (top panel) of IB4 immunofluorescence retina whole mount (p8). Graph depicting quantification of vertical branches/section analysed from 3D reconstruction (n = 6/10; p < 0.0001 unpaired students *t* test).

To study the function of Dll1 in retina angiogenesis, we generated mice with conditional loss of function of *Dll1* in myeloid cells, by crossing LysM^Cre/+^ mice to conditional alleles of *Dll1*^33^ (*Dll1*^*ΔM*^). Mice with conditional deletion of *Dll1* were born at normal Mendelian ratios and had a normal postnatal survival (Supplementary Fig. 3). However, compared to littermate controls, *Dll1* mutant mice showed increased vascular sprouting, exemplified by a significant increase in the numbers of TC at the angiogenic front and increased filopodia density at the angiogenic front and within the vascular plexus (Fig. 3A,B). Consequently, the number of vascular connection points in the superficial plexus was significantly increased at p5, a phenotype that was sustained also at p8 (Fig. 3C). Furthermore, the number of vertical vascular branches, which descend into the deeper retinal layers, was significantly higher compared to control (Fig. 3D). Together, these findings demonstrate increased angiogenesis in conditional *Dll1* mutant mice.

### Retinal myeloid cells regulate endothelial tip cell identity

**Figure 4:**
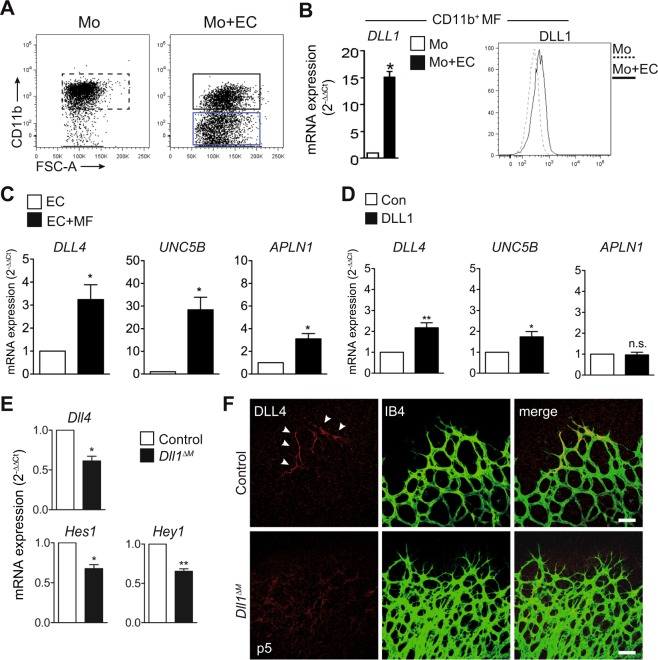
Dll1 is an upstream regulator of Dll4. (**A**) HAEC co-culture with human monocytes (CD14^+^) can be distinguished as CD11B^+^MF (Macrophages) and CD11B^−^EC (endothelial cells) after 72 hr co-culture by gating for CD11B. Monocytes (CD14^+^) and HAEC (EC) cultured alone were used as controls. Figure is representative of n > 4 independent experiments. (**B**) Real time qRT based transcriptional profiling (left) and Histogram (right) comparing DLL1 on CD14^+^ co-cultured with EC compared to CD14^+^ cultured alone. For qRT analysis, the CD14^+^ cells were re-sorted from co-culture using CD11B microbeads. 11B^+^MF (MF resorted from co-culture) were compared with CD14^+^ (cells cultured alone). n = 3 independent experiments, *p < 0.05, Students unpaired *t* test, error bars represent mean ± SEM **(C)** qRT analysis of *DLL4* and tip cells genes comparing EC’s re-sorted from co-culture using CD11B microbeads (CD11B^-^EC) with EC cultured alone (EC). n = 3 independent experiments, *p < 0.05, Students unpaired *t* test, error bars represent mean ± SEM **(D)** Transcriptional analysis by qRT depicting changes in *DLL4* and tip cell genes on HAEC cultured on DLL1 coated plates vs control. n = 4 independent experiments performed in duplicates. **p < 0.01, *p < 0.05, Students paired *t* test, error bars represent mean ± SEM **(E)** Transcriptional analysis by qRT of sorted lung ECs (CD31^+^CD144^+^) from 10 w old mice. Data mean ± SEM, n = 3/3, *p < 0.05, **p < 0.01, Students unpaired *t* test. **(F)** P5 retina whole mount immunofluorescence (50X magnification; scale bar: 50 µm).

The Notch ligand Dll4 is expressed in TC and suppresses TC formation and vessel sprouting in neighbouring stalk cells, while *Dll4* haploinsufficiency, or endothelial loss-of-function, leads to increased vascular sprouting^7,8,34,35^. In order to test the effects of Dll1 on EC we employed an *in vitro* co-culture system using human cells. Human CD14^+^ monocytes were isolated from peripheral blood and cultured *in vitro* with or without EC, which induces macrophage differentiation^11^. Subsequently, cell populations were separated by CD11b selection and analysed (Fig. 4A–C). In contrast to macrophages cultured alone, macrophages co-cultured with EC showed an upregulation of *DLL1* mRNA (Fig. 4B, left) and DLL1 protein levels (Fig. 4B, right). At the same time, EC in this co-culture displayed upregulation of *HES1* and *NRARP*, indicating active Notch signalling (Supplementary Fig. 4A). Moreover, co-cultured EC showed induction of *DLL4*, but also induction of a proto-typical tip cell genes, *UNC5B*, a vascular netrin receptor which inhibits signalling downstream of *VEGF* and *APLN1* (Fig. 4C). This demonstrates an induction of an endothelial tip cell-like signature during co-culture. To determine the response of EC to isolated Notch ligand DLL1, we cultured EC on recombinant DLL1 protein, which induced a similar degree of *HES1* induction compared to co-cultured EC (Supplementary Fig. 4B). Recombinant DLL1 induced a 2.5-fold upregulation of *DLL4* (p < 0.05), an effect size comparable to macrophage co-culture (Fig. 4D). In contrast, induction of *UNC5B* was weak, but significant (p < 0.05), while *APLN1* was not induced by recombinant DLL1. Together, these results demonstrate induction of endothelial *DLL4* by co-culture with myeloid cells and recombinant DLL1. However, culture with myeloid cells induce a more extensive tip cell program.

Furthermore, *Dll4* transcript levels were reduced in lung microvascular EC isolated from *Dll1*^*ΔM*^ mice, as were expression of the Notch target genes *Hes1* and *Hey1* (Fig. 4E), which corroborates regulation of endothelial Dll4 expression by myeloid Dll1.

We next studied Dll4 expression during vascular sprouting in the retina. Immunofluorescence confocal images of the angiogenic front in control mice revealed selective and discrete expression of Notch ligand DLL4 in tip cells, with a clear gradient between tip and stalk cells. In *Dll1*^*ΔM*^ mice, in contrast, DLL4 expression was reduced at the angiogenic front, with no discernible gradient between tip and stalk cells (Fig. 4F), thus confirming regulation of Dll4 by myeloid Dll1 in the retina.

## Discussion

In general *Dll1* haploinsufficient mice we have previously shown that the Notch ligand Dll1 regulates endothelial tip cells and vascular branching in the developing retina, but the cell source remained unknown^12^. We here demonstrate that Dll1 is expressed by a subpopulation of retinal myeloid cells, and that myeloid-specific deletion of *Dll1* increases endothelial tip cells and vascular sprouting during retinal vascular development.

The definitive identity and cellular origin of the Dll1-expressing myeloid population remains unclear, since phenotypic features and techniques that unequivocally distinguish bona fide resident microglia from monocyte-derived macrophages in the retina are only beginning to emerge^20,22,36^. However, several lines of evidence suggest that the myeloid subpopulation expressing Dll1 are monocyte-derived macrophages. First, Dll1 is selectively expressed by a population of GFP^+^IB4^hi^F4/80^hi^CD204^+^ retinal myeloid cells, a phenotypic profile displayed by monocyte-derived macrophages in the retina at baseline and after injury^21,23,28^, while GFP^+^IB4^lo^F4/80^lo^CD204^−^ myeloid cells consistent with resident microglia do not express Dll1. However, we cannot exclude that microglia activation changes expression patterns of these markers, which has been suggested for CD204 expression^37^. Second, we used a myeloid-specific targeting strategy restricted to cells of adult myelopoiesis including monocyte-derived macrophages^30^ to delete *Dll1*, which resulted in prominent vascular changes. Third, the temporal and spatial association of the myeloid cell subpopulation with expanding blood vessels in the superficial vascular plexus suggests recruitment of circulating cells. Fourth, monocyte-derived macrophages generated from isolated human monocytes *in vitro* upregulated Dll1 in co-culture with EC and influenced EC tip cell phenotype in a consistent manner. However, these arguments do not formally exclude a subpopulation of resident microglia contributing to Dll1 expression.

Conditional deletion of *Dll1* in retinal myeloid cells resulted in increased number of TC and increased filopodia, along with increased vascular density and branching, consistent with studies in tumours, in which Dll1 overexpression leads to reduced angiogenesis^38^. In contrast, general *Dll1* loss-of-function shows decreased retinal tip cell numbers and angiogenesis^12^. Besides expression in myeloid cells, Dll1 is also expressed in differentiating retinal neurons where it is necessary and sufficient to maintain a pool of progenitors in the embryonic neuroepithelium, and general *Dll1* loss-of-function leads to a significant reduction in retina size and a mild neurogenic phenotype^39^. Thus, the differences in phenotype between general and myeloid-specific *Dll1* deletion seems to reflect cell-type specific contributions of Dll1 in the developing retina, which differentially affect angiogenesis.

Previous studies have shown that myeloid cells chaperone TC fusion at the angiogenic front^13^. Our data suggest a new role for retinal myeloid cells in control of the initial steps for tip cell differentiation. A subset of retinal myeloid cells localizes to the angiogenic front and induces Dll4 expression, a Notch-dependent gene^9^, via Notch ligand Dll1. Conditional deletion of *Dll1* leads to reduced Dll4 expression in EC concomitant with increased tip cell numbers and hypersprouting *in vivo*, due to reduced Dll4 expression. This is also supported by *in vitro* findings showing induction of endothelial Dll4 by recombinant DLL1. However, compared to the effects of recombinant DLL1, macrophages co-cultured with EC *in vitro* induce a more pronounced TC signature, which suggests that tip cell fate mechanism only partially depend on Dll1. Further studies are required to address the mechanism of tip cell induction by myeloid cells.

## Methods

### Animals

The study conforms to European animal protection laws and was approved by the regional animal welfare board of Lower Saxony (LAVES). *Cx*_3_*cr1*^GFP/+^ mice (GFP^+^)^25^, *Dll1*^f/f^ mice^33^ and *LysM*^Cre/+^ mice^29^ were described previously. Gt(ROSA)26Sor mice carrying Cre-inducible lacZ alleles were obtained from The Jackson Laboratories and crossed as described^40^. Age and sex matched littermate control mice were used in all experiments. Detailed strain information is given in Supplementary Table 1.

### Retina angiogenesis model

Retina preparation and immunofluorescence of whole mounts and cryosections were performed with modifications as previously described^12^. For whole mount staining, retina were dissected from eyes and fixed overnight in 4% paraformaldehyde (PFA). The samples were blocked with PBS + 2%BSA and sequentially incubated with primary and secondary antibodies diluted in PBS/3% BSA; PBS/10% donkey serum (DLL1 staining) or PBS (DAPI) and mounted with coverslips using mounting medium (DAKO). The following antibodies or reagents were used: Isolectin-B4 (FITC, Vector, 1:100; Alexa Fluor 647; Thermo Fisher), sheep anti-DLL1 (R&D, 1:100), rat anti-F4/80 (BioLegend, 1:100), rat anti-CD204 (AbD Serotec, 1:100), anti-DLL4 (SantaCruz, 1:100), DAPI (Sigma; 1:1000). β-galactosidase staining was performed on glutaraldehyde fixed tissues as described^41^.

Stained retinae were analysed in a confocal laser scanning microscope (Leica Inverted-2, Leica Microsystems, Heidelberg) or a fluorescence microscope (Zeiss Axiovert). For 3-dimensional reconstructions confocal microscopy images of Isolectin-B4 stained whole mount retina were processed using dedicated software (Amira, Visage Imaging, Berlin). For quantitative analysis Axiovision (Zeiss, Goettingen) and ImageJ software (NIH, Bethesda) were used. TCs were identified and quantified as described^7,12^.

For qRT expression analysis, the retinas are not fixed following isolation, minced with forceps and RNA was isolated as per manufacturers instructions (RNA Nucleospin II, Macharey Nagel).

### *Ex vivo* analysis of retinal myeloid cells

The retina was harvested from unfixed mice pups. First the eye was dissected to carefully remove the retina in cold PBS (w/o Ca^2+^ and Mg^2+^). The retina was cut into small pieces and the cut pieces were incubated in an enzyme mixture containing Collagenase Type I (1250 U/ml, Worthington;CLS-1), DNase I (3000 U/ml, Sigma; DN25) and HBSS (Ca^2+^ and Mg^2+^, Biochrom AG; L2035) for 30 minutes at 37 °C. The digested mixture was filtered through a 40 µm strainer and washed with a mixture of HBSS (Ca^2+^ and Mg^2+^) containing 10% FCS and 5 mM EDTA (to arrest enzymatic activity) for 10 minutes at 1200 rpm. The resulting pellet was washed with FACS buffer once more and resuspended in FACS buffer for further staining for acquisition by flow cytometry. Cell counts were made by excluding dead cells with trypan blue. The distribution of macrophages and monocyte analysis were done by flow cytometry. After extensive washing cells were resuspended in PBS containing 10%FCS and 2 mM EDTA kept on ice, stained and used for flow cytometry.

### Flow cytometry

For *ex vivo* analysis, retinal homogenate cells were prepared by enzymatic disassociation as described previously. The cells were blocked with Trustain-FcX with the recommended protocol (101319, Biolegend) for 10 minutes. The cells were then stained with fluorochrome tagged antibodies against CD11b, F4/80, I-A/I-E, CD204 and DLL1 (Supplementary Table 3). Cells were washed twice after staining and resuspended in PBS containing 2%FCS and 2 mM Na_2_EDTA. Analysis was performed on FACS Calibur or BD-LSR II (BD Biosciences). 7AAD (1:50) was added to the culture just before analysis to exclude dead cells from the analysis.

### Isolation and *ex vivo* analysis of murine lung microvascular endothelial cells

Lungs were isolated from 10w old adult mice and cut into small pieces using sterile scalpel and washed in HBSS (without Ca^2+^/Mg^2+^, Gibco). The tissue was digested at 37 °C for 60 minutes using Dispase II (5U/ml, Invitrogen), homogenized using gentleMACS tissue dissociator (Miltenyi Biotec) and filtered. CD31^+^ cells were isolated by magnetic sorting using CD31 microbeads (Miltenyi Biotec) and further stained with CD144 antibody (BV9, Biolegend, 1:100) for sorting. CD31^+^CD144^+^ microvascular endothelial cells were sorted after excluding dead cells on FACSAria (BD Biosciences) or XDP (Beckman Coulter). RNA was isolated as per instructions (Nucleospin II, Macherey Nagel) and used for quantitative real-time PCR.

### Endothelial cell culture and co-culture experiments

Pooled Human aortic endothelial cells (HAEC) was purchased from Lonza and cultured as recommended. Confluent HAEC were split with accutase (Sigma Aldrich) and re-seeded into culture plates coated with sterile gelatine (Worthington, 0.5%, 5000cells/cm2). CD14^+^ monocytes were isolated by MACS sorting from healthy human donors (CD14^+^ microbeads, Miltenyi Biotec). HAEC were co-cultured with CD14^+^ monocytes at the density of 40,000 cells/cm2 in EBM2 (Lonza) + 1% FCS + 1% Gentamycin/Amphotericin. Re-isolation post co-culture was performed with CD11b microbeads (Miltenyi Biotec) to separate the CD11b^+^ macrophages from CD11b^−^ endothelial cells. For flow cytometry analysis of the co-cultured cells, single cell suspensions were prepared after accutase treatment and macrophages in the co-culture were analysed for DLL1 expression after gating for CD11b expression to distinguish them from CD11b^−^ EC.

### RNA analysis by real-time quantitative PCR

RNA from murine monocyte derived macrophages was isolated with the Nucleospin II (Macherey Nagel GmbH) as recommended. Reverse transcription was done using Invitrogen RT Kit (Invitrogen GmbH, Germany) according to manufacturer’s protocol. Briefly, purified template RNA added to a Master mix comprising of Buffer RT (10X), dNTP mix (5 mM each dNTP), oligo-dT primer (10 µM), RNase inhibitor (10 units/µL) and MLV Reverse Transcriptase.

For gene expression analysis, primers were designed using the software Primerquest (IDT) and PrimerBLAST (NCBI) following general guidelines for primer design. The sequence of the primers is listed in Supplementary Table 2 and used at a final concentration of 10 pmol/µL. qRT was performed using Fermentas SYBR Green PCR kit (Qiagen GmbH, Germany) as per manufacturer’s instructions. Reactions were carried out in special optical 96-well plates using a spectofluorometric thermal cycler (Lightcycler, Roche, Germany). The genes of interest and housekeeping gene were amplified using identical cycling conditions. Relative expression was calculated using standard formulas described previously^42^.

### Stimulation of endothelial cells with notch ligands

12 well plates were incubated with anti-His (Mouse monoclonal, Invitrogen) at 1 µg/mL for 30 minutes at 37 °C. The contents were aspirated and the plates were washed with PBS (Cell culture, Biochrom). The plates were incubated for 1 hour at 37 °C with DMEM + 10% FCS to prevent nonspecific binding. The blocking solution was aspirated and washed gently with PBS to remove excess blocking solution.

Recombinant DLL1 (Invitrogen, 300 µL/well, working concentration: 1 µg/mL), diluted in PBS (Biochrom, cell culture grade) was added to the wells, incubated for 2 hours at 37 °C. After incubation the contents were aspirated and washed with warm PBS (Biochrom, cell culture grade). Endothelial cells (HAEC P3) were added immediately to the wells at a density of 10000 cells/cm^2^. The cells were cultured in EBM media supplemented with the appropriate growth factors (EGM2, Lonza).

### Statistical analysis

Results are expressed as mean ± SEM. Significance of differences was calculated using unpaired, two-tailed Student’s *t* test with confidence interval of 95%. For comparison of multiple experimental groups one-way ANOVA and Bonferroni multiple-comparison test was used. p values of less than 0.05 were considered to be significant.

## Supplementary information

Supplementary Info

## Data Availability

The authors declare that all relevant data are available upon request.
