# Supplementary material for: Retinal myeloid cells regulate tip cell selection and vascular branching morphogenesis via Notch ligand Delta-like 1
Source: Sci Rep. 2019 Jul 5;9:9798. doi: 10.1038/s41598-019-46308-3 (PMC6611798; doi:10.1038/s41598-019-46308-3)
Supplement: Supplementary file 1 — Supplementary Info [file 41598_2019_46308_MOESM1_ESM.pdf]

Supplementary Information

Retinal myeloid cells regulate tip cell selection and vascular branching morphogenesis via Notch ligand Delta-like 1

Fabian Haupt, Kashyap Krishnasamy, L. Christian Napp, Michael Augustynik, Anne Limbourg, Jaba Gamrekelashvili, Johann Bauersachs, Hermann Haller, Florian P. Limbourg

Supplementary Figures

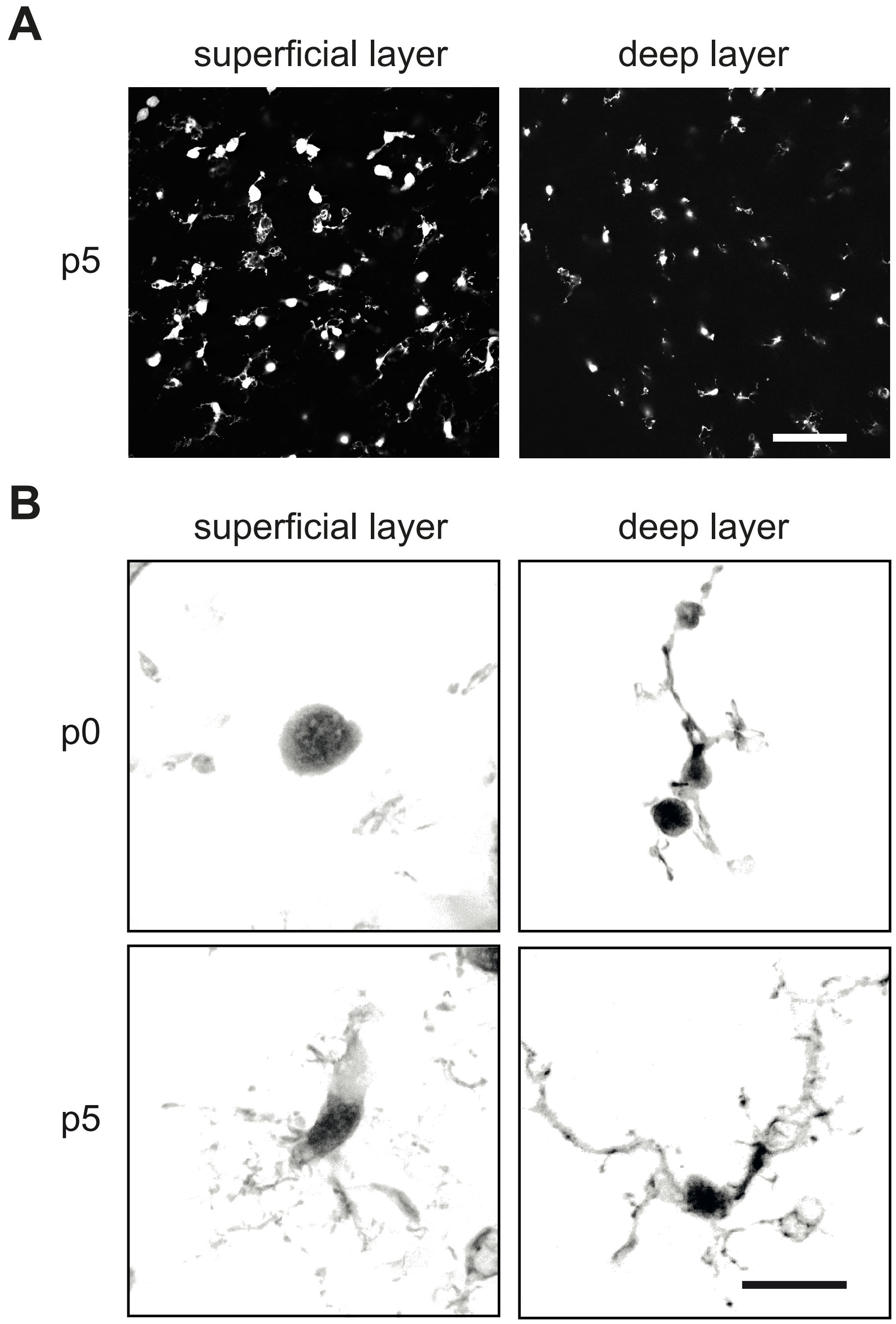

Supplementary Figure 1: Different morphologies of retinal myeloid cells (RMC)

Confocal microscopy of the GFP channel in IB4 stained (IB4 channel not shown) whole mounts of *Cx3cr1*$^{GFP/+}$ mice. **(A)** Images at p5 (50X magnification; scale bar: 75µm) with the superficial (left) and deep (right) vascular layer. **(B)** Maximum

Intensity Projections (MIP) of 4 confocal pictures (200X magnification, scale bar: 18.5 µm) of round shaped / less ramified (left) and intensively ramified RMCs (right) at p2 and p5, differentiated by their IB4 positivity.

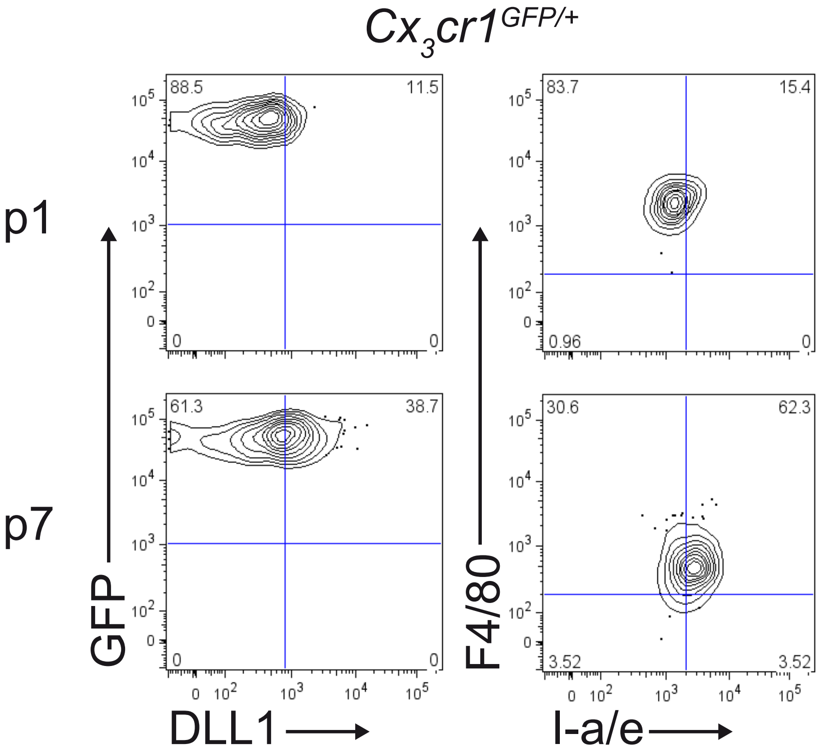

Supplementary Figure 2: RMC origin and maturation. FACS analysis of *Cx3cr1*$^{GFP/+}$ mice of p1 and p7 retinae showing histogram and contour plots of RMCs for expression of F4/80, GFP (CX3CR1) and DLL1.

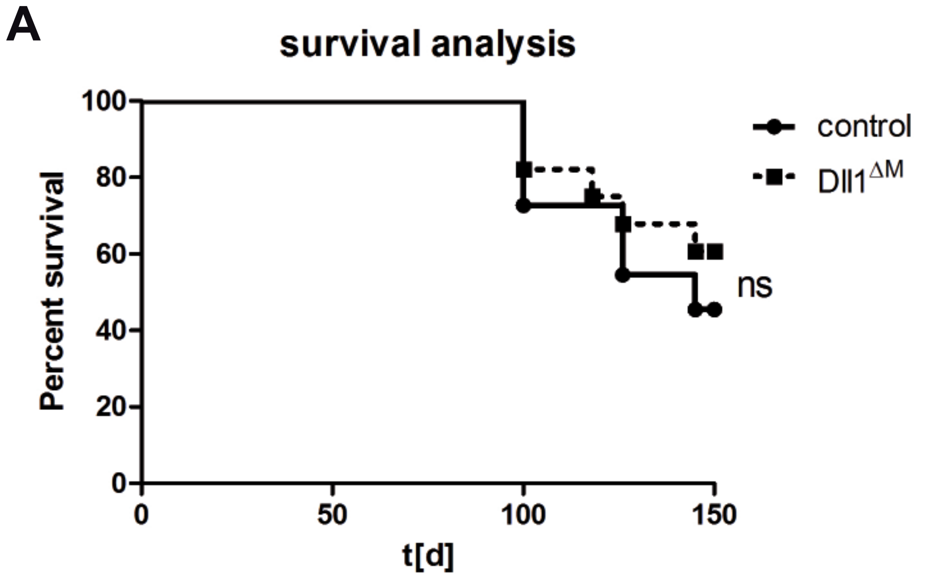

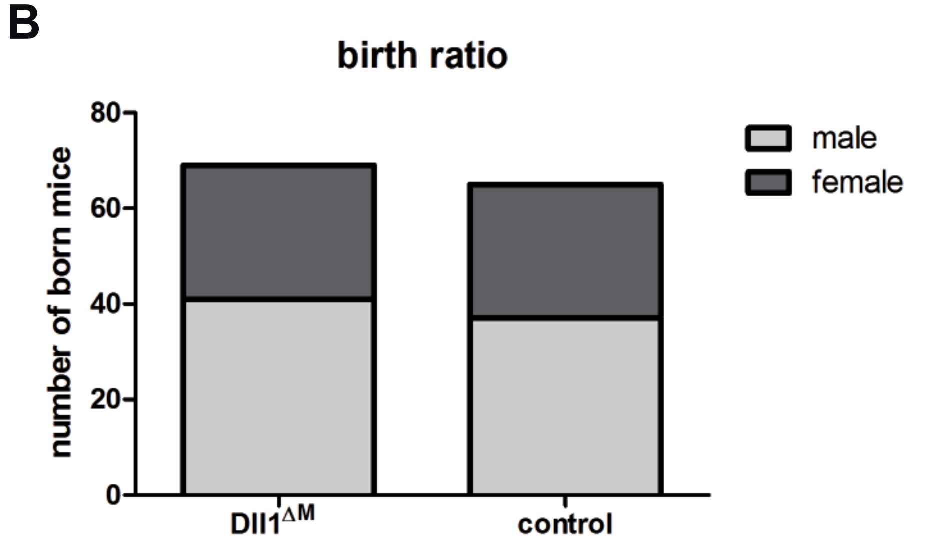

Supplementary Figure 3: Survival and birth ratio of *Dll1*$^{\Delta M}$ mice.

(A) Survival analysis of n= 28/11 newborn *Dll1*$^{\Delta M}$ vs littermate control mice over 150 days. No significant changes were detected. **(B)** Birth and gender ratio of n= 134 *Dll1*$^{\Delta M}$ and littermate control mice showing no significant differences (*Dll1*$^{\Delta M}$ 41 male vs 28 female; control 37 vs 28). Significance was defined as $p<0.05$ in Students paired *t* test.

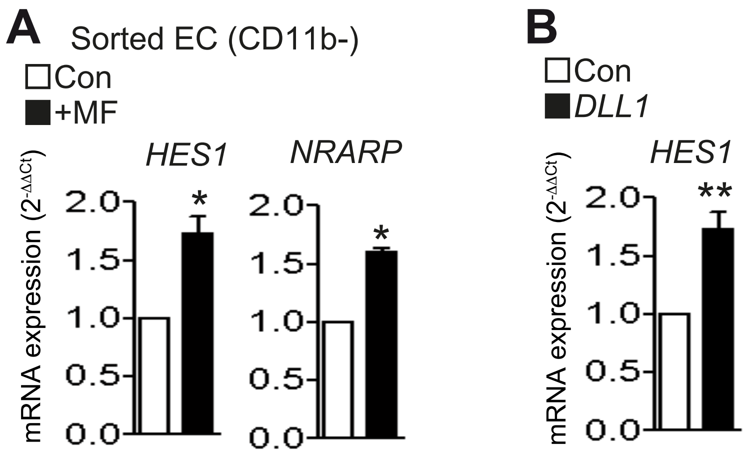

Supplementary Figure 4: Induction of Notch signalling components in human MF-endothelial cell co-culture *in vitro* **(A)** Real time PCR analysis depicting fold change in notch reporters *HES1* and *NRARP* in endothelial cells cultured alone (Con) and in co-culture (+CD14 Mo), n=3 independent experiments measured in duplicates, *p<0.05, Students paired *t* test, error bars represent mean±SEM. **(B)** Quantitative RT-PCR analysis of fold change in *HES1* in HAECs, cultured on control ligand (Con) and DLL1 Fc (DLL1), n=4 independent experiments measured in duplicates. **p<0.001, Students paired *t* test, error bars represent mean±SEM.

Supplementary tables

Supplementary Table 1: Mouse models used in the study

Name	Mouse description	Mouse background
GFP^{+}	*Cx3cr1*$^{GFP/+}$	B6
Control<br>*Dll1*$^{\Delta M}$	*LysM*$^{+/+}$*Dll1*$^{f/f}$<br>*LysM*$^{Cre/+}$*Dll1*$^{f/f}$	Mixed, B6;129<br>Mixed, B6;129
Control<br>$lacZ^{iM}$	Gt(ROSA)26Sor<br>*LysM*$^{Cre/+}$Gt(ROSA)26Sor	B6<br>B6
Control	*Dll1*$^{lox/lox}$	Mixed, B6;129

Supplementary Table 2: q-RT primers used in this study

Gene	Primer pair
Human *UNC5B*	Forward: TGG GCT GTG CAT GCA AAA TAA GAA<br>Reverse: TGC CAC GAC CAC GAA GAT GG
Human *APLN1*<br><br>Human *DLL1*	Forward: GTG TGT GGA GGG TCC CTG ATG<br>Reverse: ATT CCT TGA CCC TCT GGG CTG<br>Forward: GAG CGT GGG GAG AAA GTG TG<br>Reverse: TCT GCA CTT GCA TTC CCC TG
Human *DLL4*	Forward: ATC AGC GAT ATG CTC CCC CA<br>Reverse: TGC CTT ATA CCT CCG TGG CA
Human *NRARP*	Forward: ACA CTG CGT GGT CAA TGT GG<br>Reverse: CAG GCT GGG CGG TAT TTT CA
Human *HES1*	Forward: CAC GAC ACC GGA TAA ACC AAA G<br>Reverse: CGC GAG CTA TCT TTC TTC AGA G
Human *RPS9*	Forward: TGG TTT GCT TAG GCG CAG AC<br>Reverse: CCG CGG GGT CAC ATA AGT TT
Murine *Dll4*	Forward: GGC CGG GAA CCT TCT CAC TC<br>Reverse: TTT CCT GGC GAA GTC TCT GGC
Murine *Hes1*	Forward: CCG GAC AAA CCA AAG ACG GC<br>Reverse: GGA ATG CCG GGA GCT ATC TTT CT
Murine *Hey1*	Forward: GCG CGG ACG AGA ATG GAA AC<br>Reverse: GGC GCT TCT CGA TGA TGC CT
Murine *Rps9*	Forward: GGA TTT CTT GGA GAG GCG GC<br>Reverse: ACC TGC TTG CGG ACC CTA AT

Supplementary Table 3: Murine antibodies and fluorescence dyes for flow cytometry used in the study

Antibody	Clone	Dilution	Company
Anti-mouse/human CD11b	M1/70	1:400	BioLegend
Anti-mouse F4/80	BM8	1:100	BioLegend
Anti-mouse I-A/I-E	M5/114.15.2	1:100	Biolegend
Anti-mouse CD204	PSL204	1:100	eBioscience
Anti-human DLL1	251127	1:50	R&D Systems
Streptavidin PerCP		1:100	BD Pharmingen
7AAD		1:100	BioLegend
Propidium Iodide		1:12000	Sigma
